# Tuning adlayer-substrate interactions of graphene/h-BN heterostructures on Cu(111)–Ni and Ni(111)–Cu surface alloys†

**DOI:** 10.1039/d0ra08622c

**Published:** 2021-01-06

**Authors:** Jianmei Huang, Qiang Wang, Pengfei Liu, Guang-hui Chen, Yanhui Yang

**Affiliations:** School of Chemistry and Molecular Engineering, Institute of Advanced Synthesis (IAS), Nanjing Tech University Nanjing 211880 P. R. China wangqiang@njtech.edu.cn yhyang@njtech.edu.cn; Department of Chemistry, Shantou University Shantou Guangdong 515063 P. R. China

## Abstract

The evolution of the interface and interaction of h-BN and graphene/h-BN (Gr/h-BN) on Cu(111)–Ni and Ni(111)–Cu surface alloys *versus* the Ni/Cu atomic percentage on the alloy surface were comparatively studied by the DFT-D2 method, including the critical long-range van der Waals forces. Our results showed that the interaction strength and interface distance of Gr/h-BN/metal can be distinctly tuned by regulating the chemical composition of the surface alloy at the interface. The initially weak interaction of h-BN/Cu(111)–Ni increased linearly with increasing Ni atomic percentage, and the interface distances decreased from ∼3.10 to ∼2.10 Å. For the h-BN/Ni(111)–Cu interface, the strong interaction of the N_top_B_fcc/hcp_ stacking decreased sharply with increasing Cu atomic percentage from 0% to 50%, and the interface distances increased from ∼2.15 to ∼3.00 Å; meanwhile, the weak interaction of the B_top_N_fcc/hcp_ stacking decreased slightly with increasing Cu atomic percentage. The absorption of graphene on h-BN/Cu(111)–Ni with B_top_N_hollow_/N_top_B_fcc_ and B_top_N_hollow_/B_top_N_fcc_ stacking was more energetically favorable than that with N_top_B_hollow_/N_top_B_fcc_ and N_top_B_hollow_/B_top_N_fcc_ at Ni atomic percentages under 75%, while the interaction energy of graphene on h-BN/Cu(111)–Ni increased sharply at Ni atomic percentages higher than 75% for the B_top_N_hollow_/N_top_B_fcc_ and N_top_B_hollow_/N_top_B_fcc_ stacking. In contrast, the interaction between graphene and the h-BN/Ni(111)–Cu surface increased sharply at Cu atomic percentages lower than 25% and decreased sharply at Cu atomic percentages higher than 75%. The interaction energies were higher when the percentage of Cu atom was between 25% and 75%. The analysis of charge transfer and density of states provided further details on the changing character and evolution trends of the interactions among graphene, h-BN, and Cu–Ni surface alloy *versus* the Ni/Cu atomic percentage.

## Abstract 2

The evolution of the interface and interaction of h-BN and graphene/h-BN (Gr/h-BN) on Cu(111)–Ni and Ni(111)–Cu surface alloys *versus* the Ni/Cu atomic percentage on the alloy surface were comparatively studied by DFT-D2, including critical long-range van der Waals forces.

## Graphical abstract

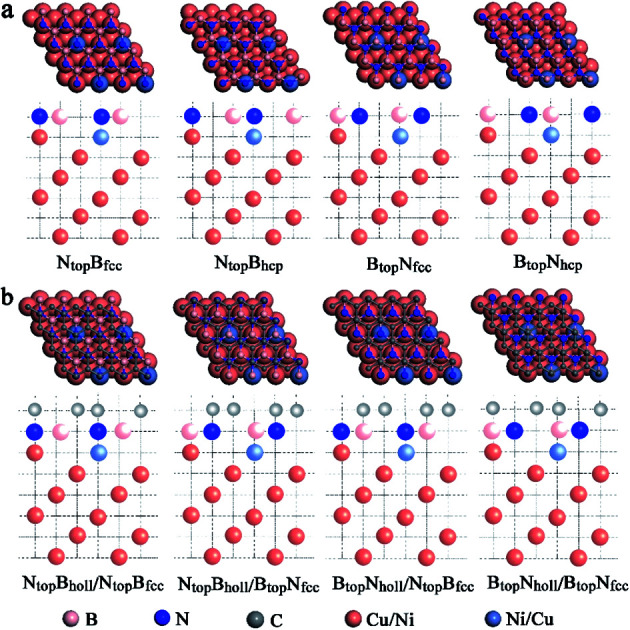

## 1. Introduction

Graphene/hexagonal boron nitride (Gr/h-BN) heterostructures have attracted much interest due to their intriguing electronic and mechanical properties.^1,2^ Great effort has been devoted to growing Gr/h-BN heterostructures with various vertically stacked or in-plane pieced patterns by chemical vapor deposition (CVD) method on various metal substrates.^2–6^ In general, Gr/h-BN heterostructures can be grown on weakly binding metal surfaces such as Cu,^1,2,7–9^ Ir,^5^ Rh,^4^ and Pt(111),^10^ resulting in a hetero-expitaxial growth mechanism of h-BN growth along the graphene edge^1,8^ or graphene nucleation at the corners of the triangular h-BN grains.^3^ In this case, the common feature of these quasi-free-standing Gr/h-BN heterostructures completely remains their intrinsic electronic properties due to the weak interfacial binding by Pauli exclusion and van der Waals (vdW) attraction.^5,10^ In contrast, Gr/h-BN heterostructures grown on strongly interacting metal substrates, such as Ni,^11,12^ Ru,^13–15^ and Re(111),^6^ result in the coexistence of perfectly patched Gr/h-BN heterostructures linked with predominant zigzag-type boundaries^6,12^ because the graphene and h-BN favor growth into separated domains. In addition, the intrinsic electronic properties of the Gr/h-BN heterostructures are almost completely inhibited due to the strong interfacial chemical bonds and charge transfer.^6^ In this regard, the complex growth mechanisms and electronic properties of the Gr/h-BN heterostructures strongly depend on the interfacial interaction among graphene, h-BN and the metal substrate. Therefore, it is highly desirable to tune the interfacial interaction strength of graphene, h-BN and the metal substrate to facilitate controllable growth and electronic properties of the Gr/h-BN heterostructure.

One possibility for tuning the interfacial interaction strength of a Gr/h-BN heterostructure is changing the chemical composition of the metal surface alloy at the interface. Recently, a single-layer Gr/h-BN in-plane heterostructure was successfully synthesized on Cu–Ni alloy substrate by a two-step low pressure CVD method.^3^ The Cu–Ni alloy substrate showed excellent catalytic performance, which not only enhanced the decomposition capability of polyaminoborane residues and the crystal quality of h-BN, but also eliminated random nucleation and promoted the growth of graphene through isothermal segregation. On the Cu–Ni alloy surface, graphene nucleated only at the top corners of the triangular h-BN grains and grew along the edge orientation of the as-formed h-BN with a fast growth rate. Subsequently, h-BN/graphene vertical stacked heterostructures were also successfully synthesized on the Cu–Ni alloy substrate by a CVD method in the same group.^16^ The interface and interactions of Gr/h-BN on pure Cu(111) and Ni(111) substrates have been studied in recent experimental and theoretical studies,^7,11,12^ which revealed the difference in the growth mechanisms and interfacial properties of the Gr/h-BN heterostructure on the weakly coupling Cu(111) and strongly interacting Ni(111) surfaces. However, few relevant experimental and theoretical investigations have been reported on the metal surface alloy, which is undoubtedly crucial to better understand the growth and electronic properties of Gr/h-BN heterostructures.

In our recent study, the interface interaction and properties of the Gr/h-BN heterostructures on pure Cu(111) and Ni(111) surfaces were investigated.^17^ The results showed that h-BN and Gr/h-BN have two typical types of interactions, weakly coupled and strong, with pure metal substrates of Cu(111) and Ni(111). The N_top_B_hcp/fcc_ stacking configurations of h-BN and Gr/h-BN on Ni(111) were strong chemisorption, while the B_top_N_hcp/fcc_ stacking configurations on Ni(111) and both N_top_B_hcp/fcc_ and B_top_N_hcp/fcc_ configurations on Cu(111) were weak physisorption. In this study, we would like to further attempt to tune the two typical interfacial interactions and surface electronic structures by regulating the chemical compositions of the surface alloys at the interface. Therefore, two types of Cu(111)–Ni and Ni(111)–Cu surface alloys with five representative Cu/Ni ratios were chosen as representatives of surface alloys to study the evolution of the interfacial interaction and properties with various Gr/h-BN heterostructures.^3,16^ The goal of this work was to elucidate the nature of the interfacial interactions and electronic properties of Gr/h-BN heterostructures on Cu–Ni surface alloys and further elaborate the influence trends of the surface alloys on the interfacial interactions and transport properties by fine-tuning the atomic percentages of Ni and Cu on the surface of Cu(111) and Ni(111).

## 2. Computational methods and models

All spin-polarized density functional theory (DFT) calculations were performed using the Vienna *Ab initio* Simulation Package (VASP).^18^ The projector augmented wave (PAW) pseudopotential^19,20^ was used for the electron–ion interactions and Perdew–Burke–Ernzerhof (PBE) generalized gradient approximation (GGA) was used for the exchange–correlation functional.^21–23^ Long-range dispersion corrections were considered within the DFT-D2 method. The dispersion coefficients *C*_6_ and vdW radii *R*_0_ for B, C, N, Ni, and Cu used in our DFT-D2 method were taken from previous work.^24,25^ The scale factor *S*_6_ was 0.75.^26,27^ The energy cutoff was set to 400 eV in the plane-wave basis set, and all calculations used a convergence criterion of 10^−6^ eV.

The pure metal surfaces were modeled by six-layer Cu(111) and Ni(111) periodic slab with the lowest two layers fixed at their equilibrium bulk phase positions, while the upper four layers were allowed to relax. The surface alloys of Cu(111)–Ni and Ni(111)–Cu were built by substituting the Cu/Ni atoms on the topmost layer of optimized p(2 × 2) Cu(111) and Ni(111) with the Ni/Cu atoms. In the surface layers of Cu(111)–Ni and Ni(111)–Cu, the number ratios of Ni to Cu or Cu to Ni atoms are 0/1, 1/3, 1/1, 3/1, and 1/0, corresponding to Ni/Cu concentrations of 0%, 25%, 50%, 75%, and 100%, respectively.

**Fig. 1:**
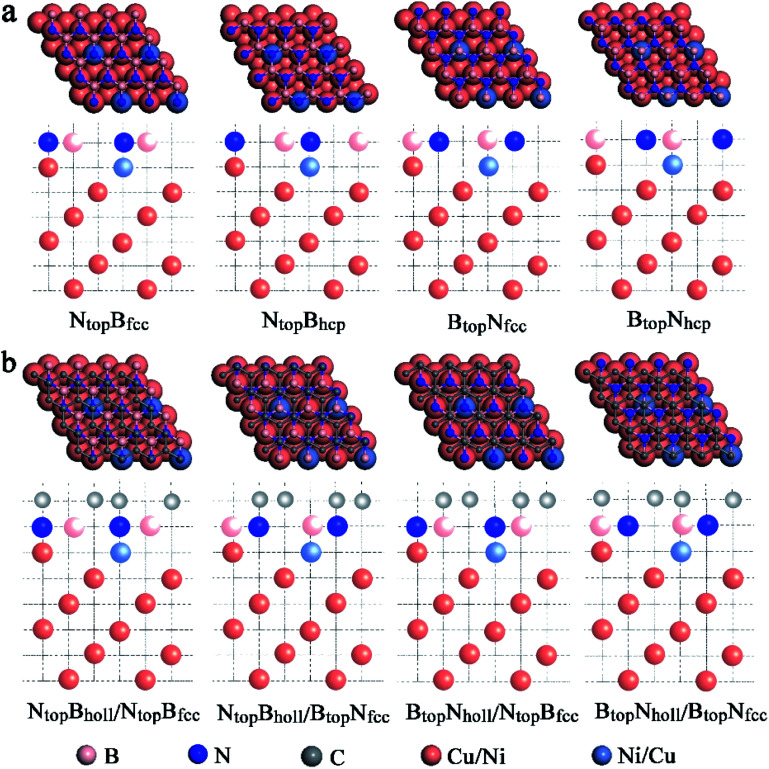
Optimized geometric structures of (a) the top and side views of monolayer h-BN with four geometries absorbed on Cu–Ni surface alloys; (b) the top and side views of monolayer graphene with four geometries absorbed on h-BN/Cu–Ni surface alloys. Pink, blue, gray, orange and purple represent B, N, C, Cu and Ni atoms, respectively.

When adsorbing the h-BN layer on the Cu(111)–Ni or Ni(111)–Cu surface alloy, there were four different configurations with B and N atoms each located at top and hollow (fcc or hcp) sites, denoted as N_top_B_fcc_, N_top_B_hcp_, B_top_N_fcc_, and B_top_N_hcp_, respectively, as shown in Fig. 1a. Furthermore, when adsorbing the graphene layer on h-BN/Cu(111)–Ni or h-BN/Ni(111)–Cu substrates, there were also four different configurations, denoted as B_top_N_hollow_/N_top_B_fcc_, N_top_B_hollow_/N_top_B_fcc_, B_top_N_hollow_/B_top_N_fcc_ and N_top_B_hollow_/B_top_N_fcc_, respectively, as shown in Fig. 1b. Each vacuum region was at least 15 Å in the direction perpendicular to the interface to avoid interactions with its own image. The Brillouin-zone integrations were performed with a 21 × 21 × 1 *k*-point mesh. It was assumed that the Gr/h-BN layers were grown on the Cu(111)–Ni and Ni(111)–Cu surface alloys. Thus, the lattice constants of Cu(111) (2.52 Å) and Ni(111) (2.45 Å) were employed in the Gr/h-BN/Cu(111)–Ni and Gr/h-BN/Ni(111)–Cu systems with lattice mismatches of 2.2% and 0.7% for the h-BN layer and 2.0% and 0.8% for the graphene layers, respectively. During the geometry optimization of Gr/h-BN/Cu(111)–Ni and Gr/h-BN/Ni(111)–Cu, the bottom two layers of the metal were fixed at their bulk lattice positions, while other layers of graphene, h-BN and metal were fully relaxed. The global transferred charges were calculated by atomic Bader charge analysis.^28,29^

## 3. Results and discussion

### 3.1. h-BN on Cu(111)–Ni and Ni(111)–Cu surfaces

#### 3.1.1. Interaction energy and interlayer distance

**Fig. 2:**
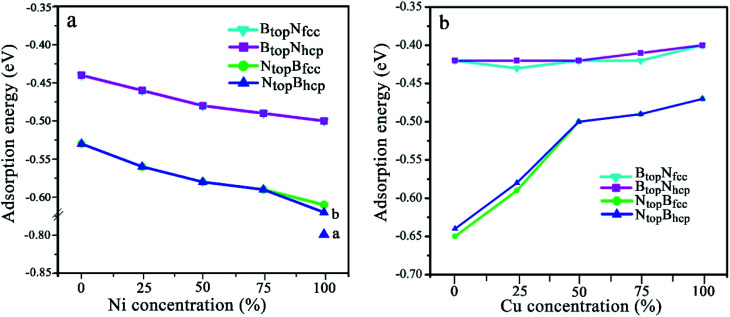
Evolution of the adsorption energy of monolayer BN on Cu(111)–Ni and Ni(111)–Cu surface alloys *versus* Ni and Cu concentration on the Cu(111)–Ni and Ni(111)–Cu alloy surface.

Four types of stable adsorption configurations of monolayer h-BN on Cu(111)–Ni and Ni(111)–Cu surface alloys are shown in Fig. 1a. The interaction energies and interlayer vertical distances are summarized in Fig. 2 and Table S1.† The interaction energies of monolayer h-BN on Cu(111)–Ni or Ni(111)–Cu surface alloys were calculated by Δ*E*_BN/M_ = (*E*_BN/M_ − (*E*_BN_ + *E*_M_))/4, where 4 is the number of super cells in each BN/M system. *E*_BN/M_, *E*_M_, and E_BN_ are the total energies of h-BN/Cu(111)–Ni or h-BN/Ni(111)–Cu, Cu(111)–Ni or Ni(111)–Cu, h-BN, respectively.

The results in Fig. 2 and Table S1† show that the interaction energies and interlayer distances of h-BN on the Cu(111)–Ni and Ni(111)–Cu surface alloys mainly depended on the Ni/Cu atomic percentages of the surface alloys and the stacking configuration. Fig. 2a shows that the interaction energy of monolayer h-BN increased linearly with increasing Ni atomic percentage on the Cu(111)–Ni alloy surface. The interaction energies of h-BN on the Cu(111)–Ni surface increased linearly from −0.53 to −0.61 eV per BN for the N_top_B_fcc_ and N_top_B_hcp_ stacking (−0.80 eV per BN for the ^a^N_top_B_fcc_ and^a^N_top_B_hcp_) and from −0.44 to −0.50 eV per BN for the B_top_N_fcc_ and B_top_N_hcp_ stacking as the Ni atomic percentages increased from 0% to 100% in the surface alloys. Accordingly, the interlayer vertical distances, *d*_BN-M_, from the h-BN layer to the topmost layer of Cu(111)–Ni decreased gradually from 2.93 to 2.71 (2.09) Å for the N_top_B_fcc_ and N_top_B_hcp_ stacking and from 3.10 to 2.95 Å for the B_top_N_fcc_ and B_top_N_hcp_ stacking. Obviously, the interaction energies of monolayer h-BN on Cu(111)–Ni with the N_top_B_fcc_ and N_top_B_hcp_ stacking were higher than those with the B_top_N_fcc_ and B_top_N_hcp_ stacking by about 0.10 eV per BN.

**Fig. 3:**
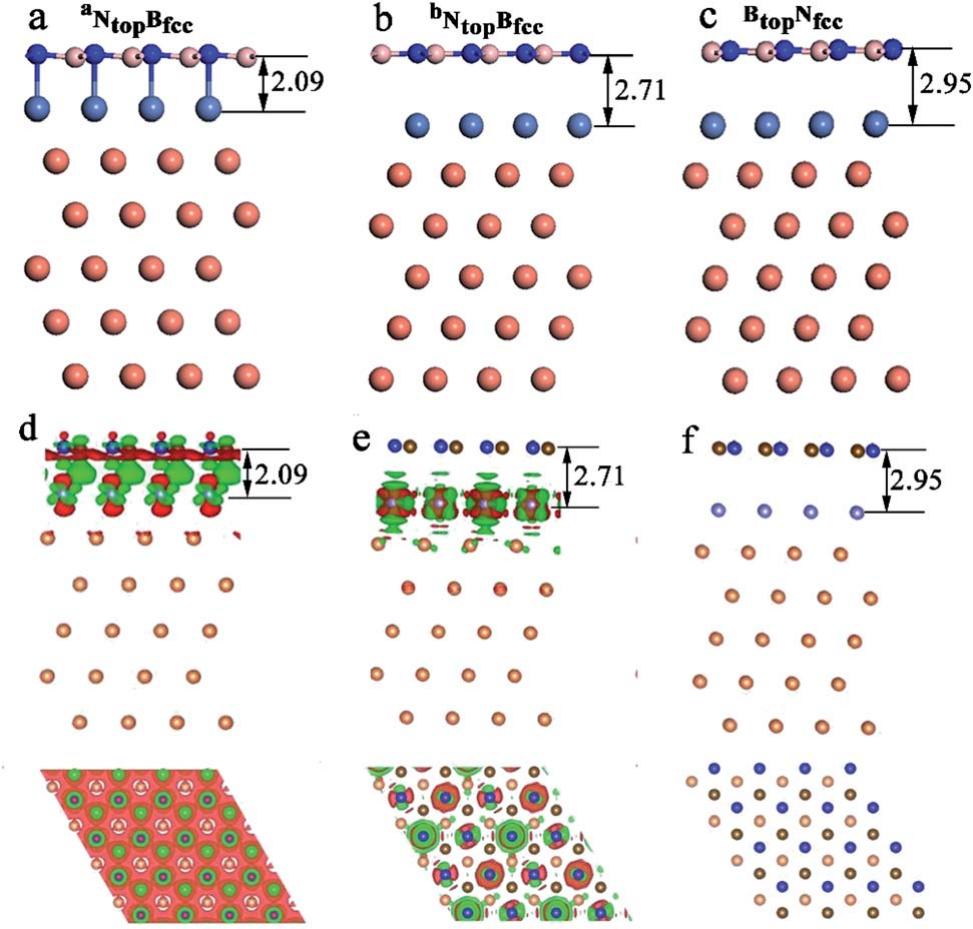
Optimized stable adsorption configurations of (a) ^a^N_top_B_fcc_, (b) ^b^N_top_B_fcc_, and (c) B_top_N_fcc_ stacking and their charge density differences (d–and f) for h-BN on Cu(111)–Ni(100%). Here, the charge density difference refers to the variance between the total charge density of h-BN/Cu(111)–Ni(100%) and the sum of the charge density of the separated Cu and Ni layer and monolayer h-BN layer, which kept the same geometric structures as those in h-BN/Cu(111)–Ni(100%). The red and green color regions mark the depletion and accumulation of electronic charges, respectively. Brown, blue, purple and orange represent B, N, Ni and Cu atoms, respectively.

Interestingly, when adsorbing monolayer h-BN on the Cu(111)–Ni(100%), there are three types of stable adsorption configurations of the ^a^N_top_B_fcc_/^a^N_top_B_hcp_, ^b^N_top_B_fcc_/^b^N_top_B_hcp_, and B_top_N_fcc_/B_top_N_hcp_ stacking, as illustrated in Fig. 2a and 3. The interaction energy profiles of h-BN adsorption in the N_top_B_fcc_ and N_top_B_hcp_ stacking geometries calculated at different interlayer distances from the h-BN layer to the topmost layer of Cu(111)–Ni clearly exhibited two stable local minima at the separations of 2.09 Å (denoted as ^a^N_top_B_fcc_/^a^N_top_B_hcp_) and 2.71/2.70 Å (denoted as ^b^N_top_B_hcp_/^b^B_top_N_fcc_), as illustrated in Fig. S2.† The differences in the interaction energies and interlayer distances between the N_top_B_fcc_ and N_top_B_hcp_ configurations were negligible for monolayer h-BN on the Cu(111)–Ni and Ni(111)–Cu alloy surfaces. For the sake of brevity, only the N_top_B_fcc_ configuration is depicted and discussed in this study. For the ^a^N_top_B_fcc_ stacking, as illustrated in Fig. 3a, the interaction energy of h-BN on Cu(111)–Ni(100%) is −0.80 eV per BN, which is significantly higher than that on pure Ni(111) by ∼0.15 eV per BN. The corresponding interlayer distance is 2.09 Å, which is shorter than that on pure Ni(111) by only 0.05 Å. However, for the ^b^N_top_B_fcc_ stacking, as illustrated in Fig. 3b, the interaction energy of h-BN on Cu(111)–Ni(100%) is about −0.61/0.62 eV per BN, which is slightly lower than that on pure Ni(111) by about 0.03 eV per BN. Meanwhile, the corresponding interlayer distance is 2.71 Å, which is longer than that on pure Ni(111) by 0.57/0.58 Å. However, the interaction energy profiles of h-BN adsorption in the B_top_N_fcc_ stacking geometries exhibit only one minimum at a separation of 2.95 Å for the weak physisorption, as illustrated in Fig. 3c. The corresponding adsorption energy is −0.50 eV per BN, which is higher than that on pure Ni(111) by only 0.08 eV per BN, and the corresponding interlayer distance is 2.95 Å, which is shorter than that on pure Ni(111) by only 0.06 Å.

On the Ni(111)–Cu surface, Fig. 2b shows that the interaction energies of h-BN on the Ni(111)–Cu surface decrease sharply from −0.65 to −0.50 eV per BN as the Cu atomic percentage increases from 0% to 50% for the N_top_B_fcc_ and N_top_B_hcp_ stacking with strong interaction. Accordingly, the interlayer vertical distances, *d*_BN-M_, from the h-BN layer to the topmost layer of Ni(111)–Cu increase gradually from 2.14 to 2.90 Å. Subsequently, the interaction energies decrease slightly from −0.50 to −0.47 eV per BN at Cu surface concentrations higher than 50%, and the interlayer distances from the h-BN layer to the Ni(111)–Cu surface only increase by about 0.06 Å. For the B_top_N_fcc_ and B_top_N_hcp_ stacking with weak interaction, the interaction energies of monolayer h-BN on the Ni(111)–Cu surface decrease slightly from −0.43 to −0.40 eV per BN as the Cu atom concentration increases from 0% to 100% on the alloy surface, and the interlayer vertical distances from the h-BN layer to the Ni(111)–Cu surface are only increased by about 0.06 Å. Similarly, the interaction energies of the N_top_B_fcc_ and N_top_B_hcp_ stacking are higher than that of the B_top_N_fcc_ and B_top_N_hcp_ stacking when the monolayer h-BN adsorbs on the Ni(111)–Cu alloy surfaces. Additionally, the difference in the adsorption energies and interlayer distances between the FCC and HCP configurations is negligible for monolayer h-BN on the Cu(111)–Ni and Ni(111)–Cu alloy surfaces.

**Fig. 4:**
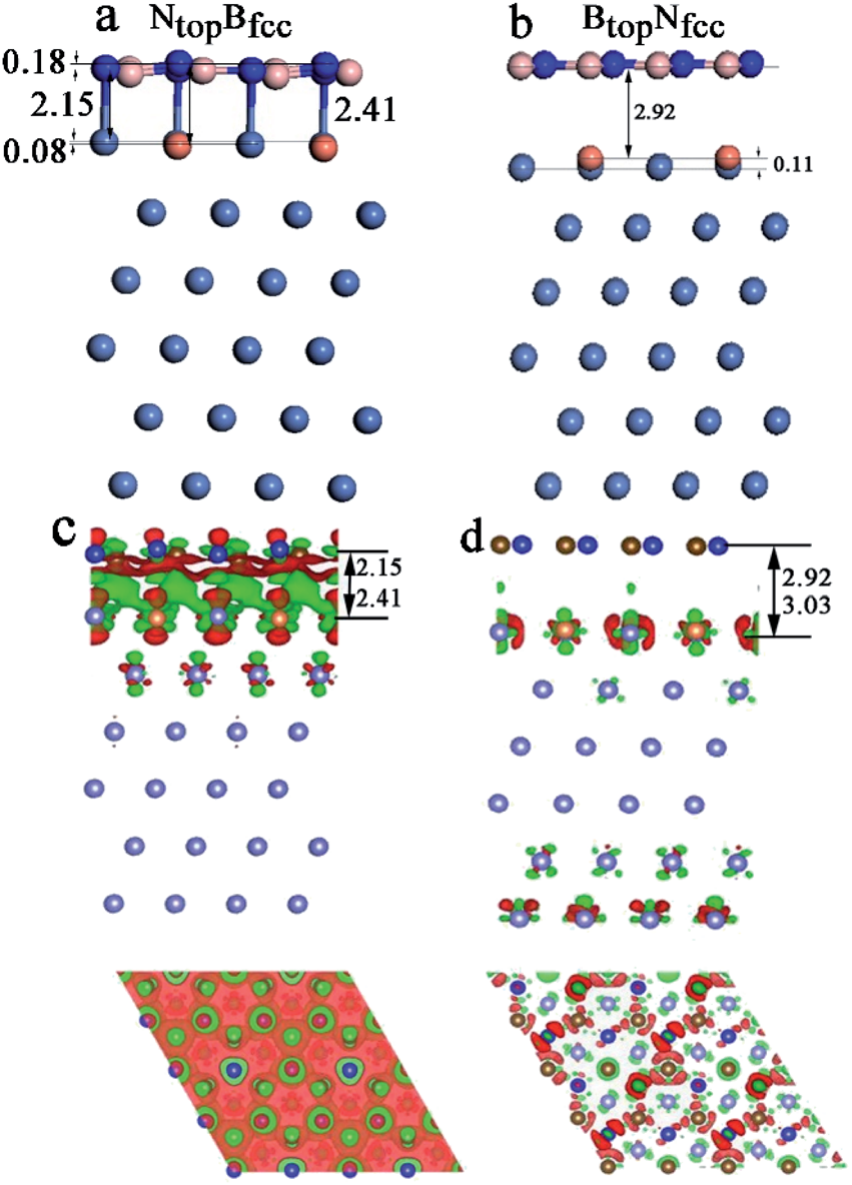
Optimized geometric structures (a and b) and charge density difference plots (c and d) of monolayer h-BN adsorbed on Ni(111)–Cu(25%). Pink, blue, gray, orange and purple represent B, N, C, Cu and Ni atoms, respectively. The positive and negative charge are shown in red and green on the charge density isosurfaces, respectively.

In addition, significant distortion occurs for the interfacial layers when adsorbing the monolayer h-BN on Ni(111)–Cu with 25% Cu, as shown in Table S1† and Fig. 4. For the N_top_B_fcc_ and N_top_B_hcp_ stacking, both the h-BN layer and topmost layer of Ni(111)–Cu afford significant distortion, as shown in Fig. 4a (only N_top_B_fcc_ stacking is shown). One N atom in h-BN bonds with the Cu atom by a longer N–Cu chemical bond of 2.41 Å, while other three N atoms of h-BN bond with the Ni atoms by shorter N–Ni chemical bonds of 2.l5 Å, as illustrated in Fig. 4a, resulting in the displacement of Cu atom downward by 0.08 Å relative to the Ni atoms in the same surface layer. Moreover, it causes the N atom bonded with Cu to move up by 0.18 Å relative to the N atoms bonded with Ni atoms. If the N atoms bonded with Ni atoms are taken as the reference, one B atom located at the Ni–Ni–Ni hollow site moves down by 0.14 Å, while three other B atoms located at the Ni–Ni–Cu hollow site are at the same height. More interestingly, for the B_top_N_fcc_ and B_top_N_hcp_ stacking, distortion occurs only at the topmost layer of Ni(111)–Cu alloys, as shown in Fig. 4b (only show B_top_N_fcc_ stacking). The Cu atoms on the Ni(111)–Cu alloy surface move up by about 0.11 Å relative to the Ni atoms in the same surface layer, while the h-BN layer is almost intact due to the relatively weak interfacial interaction, with larger interlayer distances of about 3.00 Å.

When the Cu atomic percentage is 50%, 75% or 100%, Table S1† shows that the interfacial layers become less distorted for N_top_B_fcc_ and N_top_B_hcp_ stacking because the interfacial interaction decreases with increasing interlayer vertical distance. In addition, the interaction energy and interlayer distance are not significantly different for h-BN on both the Ni(111)–Cu(100%) and pure Cu(111) surfaces. For the N_top_B_fcc_ and N_top_B_hcp_ stacking, the interaction energies of h-BN on Ni(111)–Cu(100%) are −0.47 eV per BN, which is lower than that on pure Cu(111) by 0.06 eV per BN, and the corresponding interlayer distances are ∼2.96 Å, which is longer than that on pure Cu(111) by only 0.03 Å. Differently, for the B_top_N_fcc_ and B_top_N_hcp_ stacking, the interaction energy of h-BN on Ni(111)–Cu(100%) is −0.40 eV per BN, which is lower than that on pure Cu(111) by about 0.04 eV per BN; however, the corresponding interlayer distance is ∼3.07 Å, which is shorter than that on pure Cu(111) by only 0.04–0.01 Å, respectively.

#### 3.1.2. Interfacial bonding and charge transfer

The difference in the interaction energy and interlayer distance results in a distinct difference in the interfacial properties, as illustrated in Fig. 3 and 4, which can be further validated by analyzing the charge density distribution and density of states. For the ^a^N_top_B_fcc_ stacking with strong interaction of h-BN on the Cu(111)–Ni(100%) surface, the side view in Fig. 3d shows remarkable charge transfer in the interface between h-BN and Cu(111)–Ni(100%), resulting in a chemical bond between the N atom of h-BN and the Ni atom on the Cu(111)–Ni(100%) surface. The top view in Fig. 3d shows that the charge density increases significantly at the B atoms of the h-BN layer, while it decreases at the N atoms. These results indicate that charge mainly transfers from Ni atoms to the B atoms through the N atoms of the h-BN layer. The Bader charge analysis further confirms that the N atoms have −2.12 e per atom, which is less than that in the isolated h-BN by only 0.01 e per atom, while the B atoms have +2.06 e per atom, which is more than that in the isolated h-BN by 0.07 e per atom. The surface Ni atoms and subsurface Cu atoms in Cu(111)–Ni(100%) deplete +0.02 and +0.05 e per atom, respectively. For the ^b^N_top_B_fcc_ stacking with medium interaction of h-BN on the Cu(111)–Ni(100%) surface, the side view in Fig. 3e shows that charge transfer mainly occurs in the interface between the surface Ni layer and subsurface Cu layer, while minor redistribution and electronic polarization occurs between h-BN and the surface Ni layer of Cu(111)–Ni(100%). The Bader charge analysis further confirms that the surface Ni atoms and subsurface Cu atoms in the Cu(111)–Ni(100%) deplete −0.03 and + 0.05 e per atom, respectively. The N and B atoms have −2.17 and +2.15 e per atom, which are almost equal to the charges on the N and B atoms in the isolated h-BN. For the B_top_N_fcc_ stacking with weak interaction of the h-BN on Cu(111)–Ni(100%) surface, the side view in Fig. 3f shows that there is almost no charge transfer, and only minor charge redistribution and electronic polarization occur at the interface between the h-BN layer and the Ni layer of the Cu(111)–Ni(100%) surface; less charge accumulation on the B atoms and depletion on the N atoms of the h-BN layer can be observed, as illustrated in the top view of Fig. 3f. For these Cu–Ni surface alloys, charge always transfers from Cu to Ni atoms, resulting in less charge accumulation on the Ni atoms (∼−0.02 e per atom) and charge depletion on the Cu atoms (∼+0.04 e per atom).

Similarly, for the N_top_B_fcc_ stacking with strong interfacial interaction between h-BN and Ni(111)–Cu(25%), the side and top views in Fig. 4c show that remarkable charge transfer also occurs in the interface between h-BN and Ni(111)–Cu(25%), resulting in strong chemical bonds between the N atoms of h-BN and the Ni or Cu atoms on the Ni(111)–Cu(25%) surface. The charge mainly transfers from the Ni and Cu atoms to the B atoms through the N atoms of the h-BN layer; as a result, the charge density increases significantly at the B atoms while it decreases at the N atoms of the h-BN layer, as shown in the top view of Fig. 4c. The Bader charge analysis further confirms that the B atoms have +2.10 e per atom, which is more than that in the isolated h-BN by 0.03 e per atom, and the N atoms have −2.14 e per atom, which is more than that in the isolated h-BN by only 0.01 e per atom. The surface Ni and Cu atoms and subsurface Ni atoms in Ni(111)–Cu(25%) deplete +0.02, +0.07, and +0.01 e per atom, respectively. For the B_top_N_fcc_ stacking with weak interfacial interaction between h-BN and Ni(111)–Cu(25%), there is almost no charge transfer, and only minor charge redistribution and electronic polarization at the interface between the h-BN layer and surface layer of Cu(111)–Ni(25%) occur, as illustrated in Fig. 4d.

#### 3.1.3. Partial density of states

**Fig. 5:**
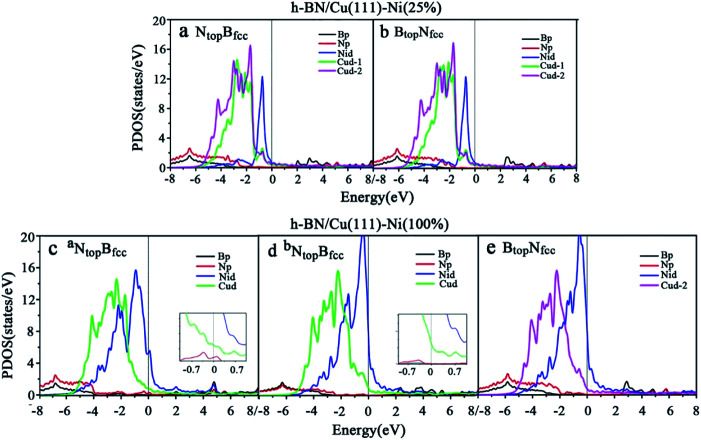
Projected density of state (PDOS) of B, N, Ni and Cu atoms for the monolayer h-BN on (a and b) Cu(111)–Ni(25%) and (c and d) Cu(111)–Ni(100%) surfaces for the N_top_B_fcc_ and (e) B_top_N_fcc_ stacking configurations.

**Fig. 6:**
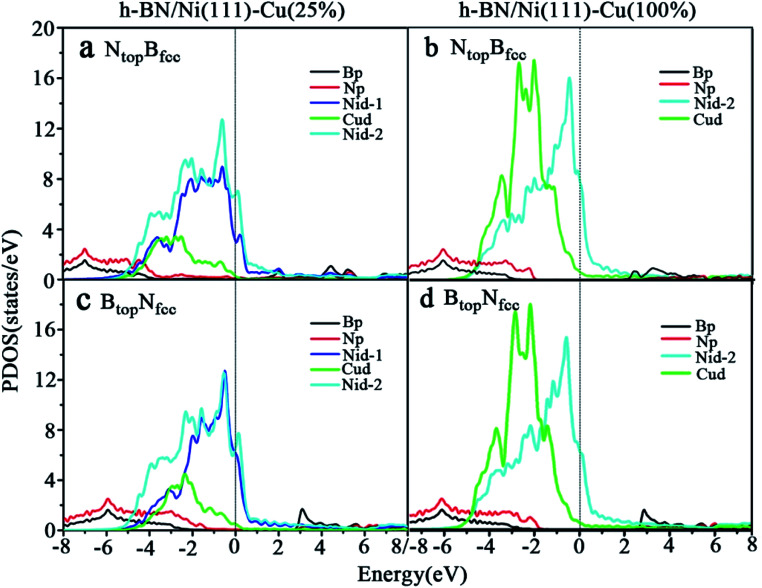
Projected densities of states (PDOS) of B, N, Ni and Cu atoms for monolayer h-BN on (a and c) Ni(111)–Cu(25%) and (b and d) Ni(111)–Cu(100%) surfaces for the (a and b) N_top_B_fcc_ and (c and d) B_top_N_fcc_ stacking configurations.

The difference in the interfacial charge transfer induces a difference in the chemical reactivity, which is correlated with the electronic structure of the interface. In order to further understand the nature of the interface bonding and the surface chemical reactivity of h-BN on Cu–Ni surface alloys, the partial densities of states (PDOS) of the B, N, Ni and Cu atoms for the monolayer h-BN on Cu(111)–Ni(25%), Cu(111)–Ni(100%), Ni(111)–Cu(25%) and Ni(111)–Cu(100%) surfaces for the N_top_B_fcc_ and B_top_N_fcc_ stacking configurations were calculated and are plotted in Fig. 5 and 6.

Compared with the pristine DOS of free h-BN in Fig. S1,† for the h-BN on Cu(111)–Ni(25%) with weak interfacial interaction, the PDOSs of the N_top_B_fcc_ and N_top_B_fcc_ stacking are similar, and non-zero charge density occurs at the Fermi level, as illustrated in Fig. 5a and b; this implies weak interfacial interactions of the π-orbitals of h-BN and the 3d orbitals of the Cu(111)–Ni(25%) surface. It can be seen that the d-states of the surface Cu atoms on Cu(111)–Ni(25%) are only slightly changed due to the embedded Ni atoms compared to those on Cu(111). However, the d-states of the Ni atoms are pronounced near the Fermi level, leading to strong overlaps with the d-states of the surface Cu atoms at −0.8 eV. This could explain the formation of the homogeneous NiCu surface alloy with strong NiCu bonding interactions.

For h-BN on Cu(111)–Ni(100%) with strong interfacial interaction, the PDOSs of the ^a^N_top_B_fcc_ stacking in Fig. 5c show higher charge densities of states around the Fermi level in comparison to the pristine PDOS of free h-BN in Fig. S1.† Frontier orbital theory indicates that a system with higher frontier electron density is chemically reactive. In this regard, the h-BN on Cu(111)–Ni(100%) with the ^a^N_top_B_fcc_ stacking would be more chemically active than the free h-BN due to the interfacial interaction and charge transfer. In addition, several new peaks appear around 4.8, 2.0, 0.2, −0.5, and −2.0 eV for the ^a^N_top_B_fcc_ stacking on the curves of the N-p and Ni-d surface states, as illustrated in Fig. 5c. These new peaks indicate strong N–Ni chemical bonding across the interface, which is caused by the hybridization between the N-p orbitals of h-BN and the 3d orbitals of Ni in the Cu(111)–Ni(100%) surface. In contrast, for the ^b^N_top_B_fcc_ and B_top_N_fcc_ stacking with medium and weak interfacial interactions, the charge densities near the Fermi level are remarkably lower than those of the ^a^N_top_B_fcc_ stacking, as shown in Fig. 5d and e; meanwhile, a few changes can still be observed on the PDOS of B, N and Ni in comparison to the pristine PDOS of free h-BN due to their interfacial interaction, indicating the weaker interfacial interaction and lower surface reactivity of the ^b^N_top_B_fcc_ and B_top_N_fcc_ stacking than of the ^a^N_top_B_fcc_ stacking.

The PDOSs of the h-BN on the Ni(111)–Cu(25%) and Ni(111)–Cu(100%) surfaces are shown in Fig. 6. In comparison to the pristine PDOS of free h-BN in Fig. S1,† the PDOSs of the N_top_B_fcc_ stacking of h-BN/Ni(111)–Cu(25%) with strong interfacial interactions show higher charge densities of states near the Fermi level, and several new peaks appear at around 5.5, 4.8, 2.2, −0.8, and −2.8 eV in the curves of N-p, Cu-d and Ni-d, as illustrated in Fig. 6a. These new peaks indicate strong N–Ni and N–Cu chemical bonds across the interface, which are caused by hybridization between the N-p orbitals of h-BN and the 3d orbitals of Ni and Cu in the Ni(111)–Cu(25%) surface. For the B_top_N_fcc_ stacking of the h-BN/Ni(111)–Cu(25%) with weak interfacial interactions, the PDOSs show a lower charge density of states near the Fermi level, and there is no new peak and orbital hybridization, as illustrated in Fig. 6c. Similarly, for both the N_top_B_fcc_ and B_top_N_fcc_ stacking of h-BN on the Ni(111)–Cu(100%) surface, there is almost zero electron density near the Fermi level and no new peak/orbital hybridization compared with the pristine PDOS of free h-BN, as shown in Fig. 6b and d, implying lower chemical reactivity and weak interactions between the π-orbitals of h-BN and the 3d orbitals of the Ni(111)–Cu(100%) surface. Overall, these results suggest that the N_top_B_fcc_ stacking of h-BN on Cu(111)–Ni(100%) and Ni(111)–Cu(25%) with charge transfer and strong interfacial interaction would be more chemically reactive than the B_top_N_fcc_ stacking of the h-BN on Cu(111)–Ni(100%) and both N_top_B_fcc_ and B_top_N_fcc_ stacking of h-BN on Ni(111)–Cu(100%). These results also imply that the chemical reactivity depends not only on the composition of the alloy surface but also on the interfacial stacking of h-BN.

### 3.2. Graphene on the h-BN/Cu(111)–Ni and h-BN/Ni(111)–Cu surfaces

#### 3.2.1. Interaction energies of 1L-Gr/h-BN with Cu(111)–Ni and Ni(111)–Cu

**Fig. 7:**
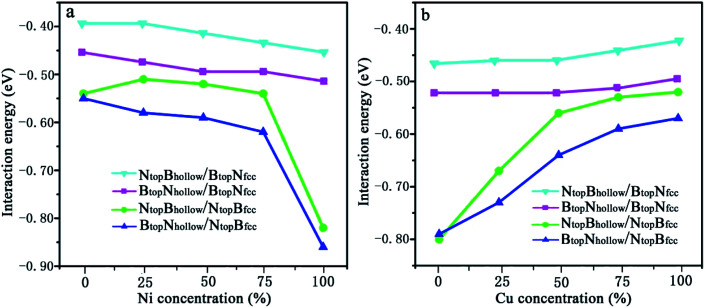
Evolution of the interfacial interactions of 1L-Gr/h-BN with Cu(111)–Ni and Ni(111)–Cu surface alloys *versus* Ni and Cu concentration on the Cu(111)–Ni and Ni(111)–Cu alloy surfaces.

**Fig. 8:**
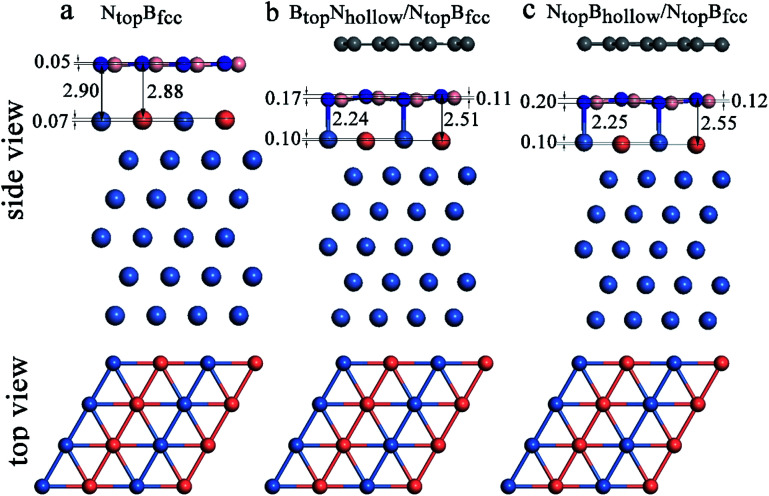
Optimized stable adsorption configurations of (a) the h-BN/Ni(111)–Cu(50%) with N_top_B_fcc_ stacking, (b) 1L-Gr/h-BN/Ni(111)–Cu(50%) with B_top_N_hollow_/N_top_B_fcc_ stacking and (c) 1L-Gr/h-BN/Ni(111)–Cu(50%) with N_top_B_hollow_/N_top_B_fcc_ stacking.

In our recent study, the interfaces and interactions of 1L-, 2L-, and 3L-Gr/h-BN heterostructures on Ni(111) and Cu(111) surfaces were examined.^17^ For the interface of the graphene and h-BN layers, the results show that the B_top_N_hollow_ stacking (C atom on top of B atom and the other on top of the hollow site of the h-BN layer) is more favorable in total energy than the N_top_B_hollow_ stacking (C atom on top of N atom and the other on top of the hollow site of the h-BN layer) and N_top_B_top_ stacking (both C atoms on top of B and N atoms of the h-BN layer). Thus, only the two more stable stacking types, B_top_N_hollow_ and N_top_B_hollow_, were considered for the Gr/h-BN interface. As mentioned above, the differences of the N_top_B_fcc_ and N_top_B_hcp_ stacking and B_top_N_fcc_ and B_top_N_hcp_ stacking for h-BN on the Cu–Ni alloy surface were negligible in their interaction energies and interlayer distances. Therefore, only the N_top_B_fcc_ and B_top_N_fcc_ stacking were considered for the interfacial stacking of the h-BN and Cu–Ni surface alloys. The optimized geometric structures of Gr/h-BN on the Cu–Ni surface alloys are displayed in Fig. 1b. The interlayer distances and interaction energies of the 1L-Gr/h-BN heterostructures with different stacking configurations on the Cu–Ni surface alloys are listed in Table S2,†Fig. 7 and 8. The interaction energy was calculated by Δ*E*_Gr/BN_ = (*E*_Gr/BN/M_ − (*E*_BN/M_ + *E*_Gr_))/4, where 4 is the number of supercells in the Gr/BN systems and *E*_Gr/BN/M_, *E*_Gr_ and *E*_BN/M_ are the total energies of the Gr/h-BN heterostructures on the alloy substrates, h-BN on the Cu–Ni alloy substrate and the freestanding graphene layer, respectively.

The results in Fig. 7 and Table S2† show that the interaction energies of Gr/h-BN/Cu–Ni depend on the Cu/Ni atomic percentage of the surface alloys and interfacial stacking configuration among graphene, h-BN and the Cu–Ni alloy surface. Similar to monolayer h-BN on Cu–Ni alloy surfaces, the interaction energies between the 1L-Gr/h-BN layer and Cu–Ni alloy surface with N_top_B_fcc/hcp_ stacking are always stronger than those with B_top_N_fcc/hcp_ stacking in 1L-Gr/h-BN/Cu–Ni. Likewise, the interaction energies between the graphene layer and h-BN/Cu–Ni substrate with B_top_N_hollow_ stacking are always stronger than that with the N_top_B_hollow_ stacking in 1L-Gr/h-BN/Cu–Ni.

The interaction energies between the 1L-Gr/h-BN and Cu–Ni alloy surfaces follow the order B_top_N_hollow_/N_top_B_fcc_ > N_top_B_hollow_/N_top_B_fcc_ > B_top_N_hollow_/B_top_N_fcc_ > N_top_B_hollow_/B_top_N_fcc_, as illustrated in Fig. 7. Fig. 7a shows that the interaction energies between the 1L-Gr/h-BN layer and Cu(111)–Ni surfaces increase significantly with increasing Ni atomic percentage on the Cu(111)–Ni alloy surface for both B_top_N_hollow_/N_top_B_fcc_ and N_top_B_hollow_/N_top_B_fcc_ stacking, while it increases slightly for both B_top_N_hollow_/B_top_N_fcc_ and N_top_B_hollow_/B_top_N_fcc_ stacking. On the weakly coupled Cu(111) surface, the interaction energies of 1L-Gr/h-BN with the pure Cu(111) surface are −0.55 and −0.54 eV per supercell for B_top_N_hollow_/N_top_B_fcc_ and N_top_B_hollow_/N_top_B_fcc_ stacking, respectively, which are almost equal to the interfacial interaction of monolayer h-BN on the Cu(111) surface. The corresponding interlayer distance of *d*_BN-M_ is 2.90 Å, which is shorter than that of monolayer h-BN on the Cu(111) surface by about 0.03 Å.

As the Ni atomic percentage increases from 0% to 75% on the Cu(111)–Ni surface layer, for the B_top_N_hollow_/N_top_B_fcc_ stacking, the interaction energies of 1L-Gr/h-BN with the Cu(111)–Ni surface increase linearly from −0.55 to −0.62 eV per supercell, as illustrated in Fig. 7a, which are comparable with those of monolayer h-BN on the corresponding Cu(111)–Ni surfaces. The corresponding interlayer distances of *d*_BN-M_ decrease linearly from 2.90 to 2.70 Å as the Ni atomic percentage increases from 0% to 75%. When the Ni atomic percentage is higher than 75%, the interaction energies of the 1L-Gr/h-BN with Cu(111)–Ni surface increase sharply from −0.62 to −0.86 eV per supercell, and the interlayer distances from h-BN layer to the Cu(111)–Ni surface decrease sharply from 2.70 to 2.07 Å.

Interestingly, for N_top_B_hollow_/N_top_B_fcc_ stacking, the interaction energies between 1L-Gr/h-BN and the Cu(111)–Ni surface decrease slightly from −0.54 to −0.51 eV per supercell, followed by an increase from −0.51 to −0.54 eV per supercell, as the Ni atomic percentage increases from 0% to 75% on the Cu(111)–Ni surface layer, as illustrated in Fig. 7a; these energies are lower than those of monolayer h-BN on the corresponding Cu(111)–Ni surfaces by about 0.05 eV per supercell. The corresponding interlayer distances of *d*_BN-M_ decrease linearly from 2.90 to 2.76 Å as the Ni atomic percentage increases from 0% to 75% for the N_top_B_hollow_/N_top_B_fcc_ stacking; these distances are shorter than those of monolayer h-BN on Cu(111)–Ni substrate by about 0.03 Å. Similarly, when the Ni atomic percentage is higher than 75%, the interaction energies of 1L-Gr/h-BN with the Cu(111)–Ni surface increase sharply from −0.54 to −0.82 eV per supercell for the N_top_B_hollow_/N_top_B_fcc_ stacking, and the interlayer distances from the h-BN layer to the Cu(111)–Ni surface decrease sharply from 2.76 to 2.07 Å.

The same change trend in the interaction energy was found for B_top_N_hollow_/B_top_N_fcc_ and N_top_B_hollow_/B_top_N_fcc_ stacking. As the Ni atomic percentage increases from 0% to 100% in the Cu(111)–Ni surface, the interaction energies between the 1L-Gr/h-BN layer and Cu(111)–Ni surface increase linearly from −0.46 to −0.51 eV per supercell for B_top_N_hollow_/B_top_N_fcc_ stacking, as illustrated in Fig. 7a, which are similar to those of monolayer h-BN on the corresponding Cu(111)–Ni surfaces. The energies also increase linearly from −0.39 to −0.45 eV per supercell for N_top_B_hollow_/B_top_N_fcc_ stacking, as illustrated in Fig. 7a, which are lower than those of B_top_N_hollow_/B_top_N_fcc_ stacking by about 0.06 eV per supercell. The interlayer distances from the h-BN layer to the Cu(111)–Ni surface, *d*_BN-M_, decrease linearly from 3.04 to 2.90 Å with increasing Ni concentration from 0% to 100% for N_top_B_hollow_/B_top_N_fcc_ and B_top_N_hollow_/B_top_N_fcc_ stacking, which are shorter than those of monolayer h-BN on the Cu(111)–Ni surface by a range of 0.02–0.06 Å.

In contrast, Fig. 7b shows that with increasing Cu atomic percentage in the Ni(111)–Cu alloy surface, the interaction energies between the 1L-Gr/h-BN layer and Ni(111)–Cu surface decrease significantly for both B_top_N_hollow_/N_top_B_fcc_ and N_top_B_hollow_/N_top_B_fcc_ stacking, while they decrease slightly for both B_top_N_hollow_/B_top_N_fcc_ and N_top_B_hollow_/B_top_N_fcc_ stacking. In comparison to monolayer h-BN on the Ni(111)–Cu alloy surface, the interaction energies between the 1L-Gr/h-BN layer and Ni(111)–Cu surface are always higher than those of the corresponding monolayer h-BN with the Ni(111)–Cu surface due to the adsorption of the upper graphene layer.

On the strong interaction Ni(111) surfaces, the B_top_N_hollow_/N_top_B_fcc_ and N_top_B_hollow_/N_top_B_fcc_ stacking of 1L-Gr/h-BN have almost identical interaction energies of −0.79 and −0.80 eV per supercell and interlayer distances of 2.13 and 2.12 Å, respectively. As the Cu atomic percentage increases from 0% to 100% on the Ni(111)–Cu surface layer, the interaction energies between 1L-Gr/h-BN and the Ni(111)–Cu surface decrease significantly from −0.79 to −0.57 eV per supercell for the B_top_N_hollow_/N_top_B_fcc_ stacking and from −0.80 to −0.52 eV per supercell for the N_top_B_hollow_/N_top_B_fcc_ stacking, as illustrated in Fig. 7b, which are higher than those of the monolayer h-BN on the corresponding Ni(111)–Cu surface (about 0.14 to 0.10 and 0.15 to 0.05 eV per supercell, respectively). The interaction energies between 1L-Gr/h-BN and the Ni(111)–Cu surface decrease slightly from −0.52 to −0.49 eV per supercell for B_top_N_hollow_/B_top_N_fcc_ stacking, as illustrated in Fig. 7b, higher than those of h-BN on the Ni(111)–Cu surface by about 0.10 eV per supercell; meanwhile, they also decrease slightly from −0.46 to −0.42 eV per supercell for N_top_B_hollow_/B_top_N_fcc_ stacking, as illustrated in Fig. 7b, which are also higher than those of monolayer h-BN on the corresponding Ni(111)–Cu surface by about 0.03 eV per supercell but lower than those of the B_top_N_hollow_/B_top_N_fcc_ stacking by about 0.07 eV per supercell.

Interestingly, when graphene adsorbs on h-BN/Ni (111)–Cu(50%), the interlayer distances between the h-BN layer and the Ni(111)–Cu(50%) surface are reduced from 2.88^Cu^/2.90^Ni^ to 2.51^Cu^/2.24^Ni^ Å for B_top_N_hollow_/N_top_B_fcc_ stacking and from 2.88^Cu^/2.90^Ni^ to 2.55^Cu^/2.25^Ni^ Å for N_top_B_hollow_/N_top_B_fcc_ stacking, as illustrated in Fig. 8, Tables S1 and S2.† The corresponding interaction energies between 1L-Gr/h-BN and the Ni(111)–Cu(50%) surface increase from −0.50 to −0.64 eV per supercell for B_top_N_hollow_/N_top_B_fcc_ stacking and from −0.50 to −0.56 eV per supercell for N_top_B_hollow_/N_top_B_fcc_ stacking. However, for B_top_N_hollow_/B_top_N_fcc_ and N_top_B_hollow_/B_top_N_fcc_ stacking in 1L-Gr/h-BN/Ni(111)–Cu(50%) and all other stacking configurations in 1L-Gr/h-BN/Ni(111)–Cu(>50%), Table S1† shows that the interfacial layers become less distorted due to the relatively weak interaction energies and large interlayer distances. Only the topmost layer of the Ni(111)–Cu alloy shows noticeable distortion, in which the Cu atoms move up by about 0.10 Å relative to the Ni atoms in same surface layer, while the h-BN layer is almost unchanged.

#### 3.2.2. Interaction energies of monolayer graphene with h-BN/Cu(111)–Ni and h-BN/Ni(111)–Cu

**Fig. 9:**
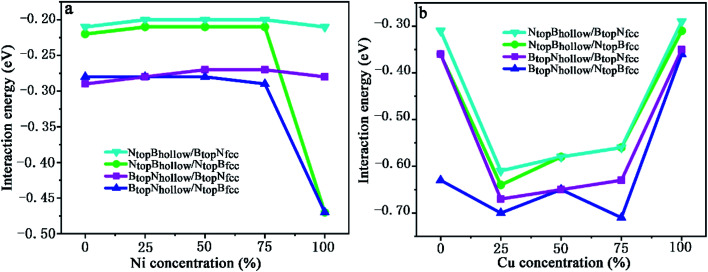
Evolution of the interfacial interaction of 1L-Gr with h-BN/Cu(111)–Ni and h-BN/Ni(111)–Cu surface alloys *versus* Ni and Cu concentration on the Cu(111)–Ni and Ni(111)–Cu alloy surface.

In this section, the interlayer interactions between the graphene layer and h-BN/Cu(111)–Ni and h-BN/Ni(111)–Cu are further discussed comparatively. The interaction energies between the graphene layer and the h-BN/Cu(111)–Ni and h-BN/Ni(111)–Cu surface are shown in Fig. 9, calculated by Δ*E*_Gr/BN_ = (*E*_Gr/BN/M_ − *E*_BN/M_ − *E*_Gr_)/4, where 4 is the number of supercells in the Gr/BN systems and *E*_Gr/BN/M_, *E*_BN/M_, and *E*_Gr_ are the total energies of Gr/h-BN/M(111)-N, h-BN/M(111)-N, and graphene, respectively.

When the Ni atomic percentage is less than 75% on the Cu(111)–Ni alloy surface, the interaction energy between the graphene layer and h-BN/Cu(111)–Ni surface only depends on the stacking configuration of graphene and the h-BN layers. Fig. 9a shows that the interaction energies between graphene and the h-BN/Cu(111)–Ni surface range from −0.28 to −0.29 eV per supercell for the B_top_N_hollow_/N_top_B_fcc_ and B_top_N_hollow_/B_top_N_fcc_ stacking, which are slightly higher than those of the N_top_B_hollow_/N_top_B_fcc_ and N_top_B_hollow_/B_top_N_fcc_ stacking by about 0.06 eV per supercell. For N_top_B_hollow_/N_top_B_fcc_ and N_top_B_hollow_/B_top_N_fcc_ stacking, the interaction energies between graphene and the h-BN/Cu(111)–Ni surface range from −0.22 to −0.21 eV per supercell, which are comparable with those of L-Gr/h-BN without metal substrates.

When the Ni atomic percentage is higher than 75% on the Cu(111)–Ni alloy surface, the interaction energy between graphene and the h-BN/Cu(111)–Ni surface depends not only on the stacking configuration of the graphene and h-BN, but also on the stacking configuration of h-BN and Cu(111)–Ni. Fig. 9a shows that the interaction energy between graphene and h-BN/Cu(111)–Ni increases sharply from −0.29 to −0.47 eV per supercell for B_top_N_hollow_/N_top_B_fcc_ and from −0.21 to −0.47 eV per supercell for N_top_B_hollow_/N_top_B_fcc_ stacking as the Ni atomic percentage increases from 75% to 100% on the Cu(111)–Ni alloy surface; this can be attributed to the strong interaction of the N_top_B_fcc_ stacking of h-BN and the Cu(111)–Ni surface when the Ni surface atomic percentage is higher than 75%. The interaction energies between graphene and h-BN/Cu(111)–Ni remain at about −0.28 eV per supercell for the B_top_N_hollow_/B_top_N_fcc_ stacking and about −0.21 eV per supercell for the N_top_B_hollow_/B_top_N_fcc_ stacking due to the weak interaction of B_top_N_fcc_ stacking of h-BN with the Cu(111)–Ni surface with a higher Ni surface atomic percentage.

On the Ni(111)–Cu alloy surface, the interaction energies between graphene and the h-BN/Ni(111)–Cu surface are distinctly higher than those of the corresponding freestanding Gr/h-BN. Interestingly, when the Cu atomic percentage is less than 25% on the Ni(111)–Cu alloy surface, Fig. 9b shows that the interaction energies between graphene and the h-BN/Ni(111)–Cu surface increase sharply from −0.31 to −0.61 eV per supercell for N_top_B_hollow_/B_top_N_fcc_ stacking, from −0.36 to −0.64 eV per supercell for N_top_B_hollow_/N_top_B_fcc_ stacking, and from −0.36 to −0.67 eV per supercell for B_top_N_hollow_/B_top_N_fcc_ stacking, while it increases slightly from −0.63 to −0.70 eV per supercell for B_top_N_hollow_/N_top_B_fcc_ stacking.

With increasing Cu atomic percentage from 25% to 75% on the Ni(111)–Cu alloy surface, Fig. 9b shows that the interaction energies between the graphene layer and h-BN/Cu(111)–Ni surface decrease linearly from −0.61 to −0.56 eV per supercell for N_top_B_hollow_/B_top_N_fcc_, from −0.64 to −0.56 eV per supercell for N_top_B_hollow_/N_top_B_fcc_ and from −0.67 to −0.63 eV per supercell for B_top_N_hollow_/B_top_N_fcc_. The interaction energy of B_top_N_hollow_/N_top_B_fcc_ decreases slightly from −0.70 to −0.71 eV per supercell, followed by an increase from −0.65 to −0.71 eV per supercell as the Cu atomic percentage increases from 50% to 75%. However, the interaction energies between graphene and the h-BN/Cu(111)–Ni surface decrease sharply from −0.56 to −0.29 eV per supercell for N_top_B_hollow_/B_top_N_fcc_ stacking, from −0.56 to −0.31 eV per supercell for N_top_B_hollow_/N_top_B_fcc_ stacking, from −0.63 to −0.35 eV per supercell for B_top_N_hollow_/B_top_N_fcc_ stacking and from −0.71 to −0.36 eV per supercell for B_top_N_hollow_/N_top_B_fcc_ stacking as the Cu atomic percentage increases from 75% to 100% on the Ni(111)–Cu alloy surface.

The charge density difference of 1L-Gr/h-BN/Cu(111)–Ni and 1L-Gr/h-BN/Ni(111)–Cu is plotted in Fig. S4.† Regarding all the interfaces, the interface between h-BN with Cu(111)–Ni and Ni(111)–Cu substrates shows similar charge transfer and charge accumulation/polarization to that in the h-BN/Cu(111)–Ni and h-BN/Ni(111)–Cu, as shown in Fig. S3.† Furthermore, the interface between the graphene layer and h-BN affords neither charge accumulation nor electron polarization in 1L-Gr/h-BN/Cu(111)–Ni and 1L-Gr/h-BN/Ni(111)–Cu. The results indicate that the adsorption of monolayer graphene on h-BN/Cu(111)–Ni and h-BN/Ni(111)–Cu is physisorption by weak interfacial binding of Pauli exclusion and van der Waals forces, implying that the intrinsic electronic properties of graphene would be completely maintained when growing graphene layers on these h-BN/Cu(111)–Ni or h-BN/Ni(111)–Cu substrates.

## 4. Discussion

Recent experimental and theoretical studies have indicated that the interfacial interaction strength among graphene, h-BN, and the metal substrate plays important roles in the controllable growth and electronic properties of Gr/h-BN heterostructures.^4–6^ Experimental studies have reported that Gr/h-BN growth on strongly interacting metal substrates results in the coexistence of perfectly patched Gr/h-BN heterostructures linked with predominant zigzag-type boundaries,^6,12^ while the Gr/h-BN growth on the weakly binding metal surfaces leads to a hetero-expitaxial growth mechanism of h-BN growth along the graphene edge^1^ or graphene nucleation at the corners of the triangular h-BN grains.^3^ It is important to be able to tune the interaction among graphene, h-BN, and the metal surface at different growth stages to achieve controlled growth, which can be achieved through doping metal surfaces to form metal alloys.

The intention of this work was to find a general guideline for the selection and tuning of metal surface alloys for controlled growth of Gr/h-BN and also to provide some understanding of the graphene-h-BN-metal interface for potential electronic device design. Thus, we further extended our study to investigate the interfacial structures and interactions of Gr/h-BN layers with the bimetallic Ni/Cu(111) surface alloys, and we focused on the overall interaction among large graphene, h-BN layer and metal surface alloys in addition to the individual steps at different Gr/h-BN growth stages. Our results from the interaction energy, geometric structure, charge transfer, and DOS analyses demonstrate that the interaction strength among graphene, h-BN, and the bimetallic alloy surface can be tuned selectively by reasonably regulating the atomic percentage on the alloy surface. The initially weak interfacial interaction of h-BN/Cu(111) can be enhanced substantially by introducing a Ni surface. In contrast, the initially strong interfacial interaction of h-BN/Ni(111) can be reduced successfully by introducing a Cu surface.

Recently, Lu *et al.*^3^ demonstrated successful CVD synthesis of controlled growth of high-quality Gr/h-BN in-plane and stacked heterostructures on Cu–Ni alloy. They verified that the introduction of nickel to a copper substrate not only enhances the catalytic capability of decomposing polyaminoborane residues but also promotes graphene growth *via* isothermal segregation. Based on the findings from this study, we proposed a new strategy to control growth of high-quality Gr/h-BN, that is, to design surface alloy catalysts by synergetic combination of the distinct catalytic capabilities of Cu and Ni and the well-known segregation phenomenon in Cu/Ni binary alloy. The Ni (Cu) atoms introduced to the surface or subsurface layers of Cu (Ni) substrates can be used as high catalytic activity sites, while the Cu atoms located at the surface or subsurface cam be employed as a favorable segregation medium, such as Cu(111)–Ni–Cu (Ni(111)–Ni–Cu). To facilitate controllable growth of Ge/h-BN, this can be achieved through regulating the atomic percentage and thickness of the Ni–Cu surface layers in the Cu(111)–Ni–Cu or Ni(111)–Ni–Cu surface alloys; therefore, the catalytic capability and isothermal segregation of boron-nitride and carbon species on the Cu(111)–Ni–Cu or Ni(111)–Ni–Cu surface layers cam be changed to provide the desired growth conditions.

## 5. Conclusions

The evolution of the interface and interaction of the monolayer h-BN and 1L-Gr/h-BN heterostructures on Cu(111)–Ni and Ni(111)–Cu surface alloys *versus* Ni/Cu atomic percentage were comparatively studied by the DFT-D2 method. For monolayer h-BN on both Cu(111)–Ni and Ni(111)–Cu, the interaction energies of the N_top_B_fcc/hcp_ stacking are higher than those of the B_top_N_fcc/hcp_ stacking. The interaction energy of h-BN on the Cu(111)–Ni surface increased linearly with increasing Ni atomic percentage for these four stacking configurations. In contrast, the interaction energies of h-BN on Ni(111)–Cu decreased slightly as the Cu atomic percentage increased, except that the interaction energies decreased sharply as the Cu atomic percentage increased from 0% to 50% for the N_top_B_fcc/hcp_ stacking. The interlayer distances between h-BN and Cu(111)–Ni decreased gradually with increasing Ni atomic percentage and increased gradually with increasing Cu atomic percentage for h-BN and Ni(111)–Cu.

The interaction strength of the 1L-Gr/h-BN heterostructure on Cu(111)–Ni and Ni(111)–Cu followed the order B_top_N_hollow_/N_top_B_fcc_ > N_top_B_hollow_/N_top_B_fcc_ > B_top_N_hollow_/B_top_N_fcc_ > N_top_B_hollow_/B_top_N_fcc_. For both B_top_N_hollow_/N_top_B_fcc_ and N_top_B_hollow_/N_top_B_fcc_ stacking, the interaction energies of 1L-Gr/h-BN on Cu(111)–Ni increased sharply when the Ni atomic percentage was higher than 75%, while the interaction energies of 1L-Gr/h-BN on Ni(111)–Cu decreased significantly when the Cu atomic percentage was higher than 50%. However, for the N_top_B_hollow_/B_top_N_fcc_ and B_top_N_hollow_/B_top_N_fcc_ stacking, the interaction energies changed only slightly for 1L-Gr/h-BN on Cu(111)–Ni and Ni(111)–Cu. The interaction energy of graphene on h-BN/Cu(111)–Ni with B_top_N_hollow_ stacking were higher than that with N_top_B_hollow_ stacking at Ni atomic percentages under 75%, while the interaction energy of graphene on h-BN/Cu(111)–Ni increased sharply at Ni atomic percentages higher than 75% for the N_top_B_hollow_/N_top_B_fcc_ and B_top_N_hollow_/N_top_B_fcc_ stacking. Differently, the interaction energies between graphene and the h-BN/Ni(111)–Cu surface increased sharply at Cu atomic percentages lower than 25%, while they decreased sharply at Cu atomic percentages higher than 75%. The interaction energies were higher when the percentage of Cu atom was between 25% and 75%. These results suggest that the interfacial interactions and the properties of graphene, h-BN and Cu–Ni alloy can be regulated by tuning the Cu/Ni atomic percentage on the Cu–Ni surface alloys, which provides insight into the epitaxial growth of Gr/h-BN heterostructures and the design of Gr/h-BN-based electronic devices.

## Conflicts of interest

The authors declare that there are no conflicts of interest.

## Supplementary Material

RA-011-D0RA08622C-s001

## References

[cit1] Liu L., Park J., Siegel D. A., McCarty K. F., Clark K. W., Deng W., Basile L., Idrobo J. C., Li A. P., Gu G. (2014). Science.

[cit2] Song X. J., Gan T., Nie Y. F., Zhuang J. N., Sun J. Y., Ma D. L., Shi J. P., Lin Y. W., Ding F., Zhang Y. F., Liu Z. F. (2016). Nano Lett..

[cit3] Lu G. Y., Wu T. R., Yang P., Yang Y. C., Jin Z. H., Chen W. B., Jia S., Wang H. M., Zhang G. H., Sun J. L., Ajayan P. M., Lou J., Xie X. M., Jiang M. H. (2017). Adv. Sci..

[cit4] Gao Y. B., Zhang Y. F., Chen P. C., Li Y. C., Liu M. X., Gao T., Ma D. L., Chen Y. B., Cheng Z. H., Qju X. H., Duan W. H., Liu Z. F. (2013). Nano Lett..

[cit5] Liu M. X., Li Y. C., Chen P. C., Sun J. Y., Ma D. L., Li Q. C., Gao T., Gao Y. B., Cheng Z. H., Qiu X. H., Fang Y., Zhang Y. F., Liu Z. F. (2014). Nano Lett..

[cit6] Qi Y., Han N., Li Y., Zhang Z., Zhou X., Deng B., Li Q., Liu M., Zhao J., Liu Z., Zhang Y. (2017). ACS Nano.

[cit7] Gao T., Song X., Du H., Nie Y., Chen Y., Ji Q., Sun J., Yang Y., Zhang Y., Liu Z. (2015). Nat. Commun..

[cit8] Han G. H., Rodriguez-Manzo J. A., Lee C.-W., Kybert N. J., Lerner M. B., Qi Z. J., Dattoli E. N., Rappe A. M., Drndic M., Johnson A. T. C. (2013). ACS Nano.

[cit9] Meng J. H., Zhang X. W., Wang H. L., Ren X. B., Jin C. H., Yin Z. G., Liu X., Liu H. (2015). Nanoscale.

[cit10] Nappini S., Pis I., Mentes T. O., Sala A., Cattelan M., Agnoli S., Bondino F., Magnano E. (2016). Adv. Funct. Mater..

[cit11] Oshima C., Itoh A., Rokuta E., Tanaka T., Yamashita K., Sakurai T. (2000). Solid State Commun..

[cit12] Drost R., Kezilebieke S., Ervasti M. M., Hamalainen S. K., Schulz F., Harju A., Liljeroth P. (2015). Sci. Rep..

[cit13] Lu J., Gomes L. C., Nunes R. W., Castro Neto A. H., Loh K. P. (2014). Nano Lett..

[cit14] Sutter P., Huang Y., Sutter E. (2014). Nano Lett..

[cit15] Sutter P., Cortes R., Lahiri J., Sutter E. (2012). Nano Lett..

[cit16] Lu G. Y., Zhang G. H., Sun J. L., Wang X. J., Shi Z. Y., Jiang D., Wang H. M., Li A., Wu T. R., Yu Q. K., Xie X. M. (2019). Carbon.

[cit17] Wang Q., Liu P., Bian X., Huang J., Li W.-q., Chen G.-h., Yang Y. (2019). Appl. Surf. Sci..

[cit18] Kresse G., Furthmuller J. (1996). Phys. Rev. B: Condens. Matter Mater. Phys..

[cit19] Kresse G., Joubert D. (1999). Phys. Rev. B: Condens. Matter Mater. Phys..

[cit20] Blochl P. E. (1994). Phys. Rev. B: Condens. Matter Mater. Phys..

[cit21] Perdew J. P., Burke K., Wang Y. (1996). Phys. Rev. B: Condens. Matter Mater. Phys..

[cit22] Fuentes-Cabrera M., Baskes M. I., Melechko A. V., Simpson M. L. (2008). Phys. Rev. B: Condens. Matter Mater. Phys..

[cit23] Perdew J. P., Burke K., Ernzerhof M. (1996). Phys. Rev. Lett..

[cit24] Grimme S. (2006). J. Comput. Chem..

[cit25] Bucko T., Hafner J., Lebegue S., Angyan J. G. (2010). J. Phys. Chem. A.

[cit26] Hamada I., Otani M. (2010). Phys. Rev. B: Condens. Matter Mater. Phys..

[cit27] Vanin M., Mortensen J. J., Kelkkanen A. K., Garcia-Lastra J. M., Thygesen K. S., Jacobsen K. W. (2010). Phys. Rev. B: Condens. Matter Mater. Phys..

[cit28] Sanville E., Kenny S. D., Smith R., Henkelman G. (2007). J. Comput. Chem..

[cit29] Henkelman G., Arnaldsson A., Jonsson H. (2006). Comput. Mater. Sci..

