# Supplementary material for: Tuning adlayer-substrate interactions of graphene/h-BN heterostructures on Cu(111)–Ni and Ni(111)–Cu surface alloys
Source: RSC Adv. 2021 Jan 6;11(4):1916–27. doi: 10.1039/d0ra08622c (PMC8693814; doi:10.1039/d0ra08622c)
Supplement: RA-011-D0RA08622C-s001 [file RA-011-D0RA08622C-s001.pdf]

Electronic Supplementary Material (ESI) for RSC Advances.
This journal is © The Royal Society of Chemistry 2020

Supporting Information

Tuning Adlayer-substrate Interactions of Graphene/h-BN Heterostructures on Cu(111)-Ni and Ni(111)-Cu Surface Alloys

*Qiang Wang**, [a], *Jianmei Huang*[a], *Pengfei Liu*[a], Guang-hui Chen[b], *and Yanhui Yang**, [a]

[a]School of Chemistry and Molecular Engineering, Institute of Advanced Synthesis (IAS), Nanjing Tech University, Nanjing 211880, P. R. China

[b]Department of Chemistry, Shantou University, Guangdong, Shantou, 515063, P. R. China

To whom correspondence should be addressed:

Email: wangqiang@njtech.edu.cn (Qiang Wang)

Email: yhyang@njtech.edu.cn (Yanhui Yang)

Table S1. The interaction energy ($\Delta E_{BN/M}$, eV/BN) and interlayer vertical distance (d_{BN-M}, Å) of monolayer h-BN on the Cu(111)-Ni and Ni(111)-Cu surface alloys with four stable adsorption configurations. Adsorption configurations correspond to the illustration in Figure 1a.

Substrate	$N_{top}B_{fcc}$		$N_{top}B_{hcp}$		$B_{top}N_{fcc}$		$B_{top}N_{hcp}$	
	$\Delta E_{BN/M}$	d_{BN-M}	$\Delta E_{BN/M}$	d_{BN-M}	$\Delta E_{BN/M}$	d_{BN-M}	$\Delta E_{BN/M}$	d_{BN-M}
Cu(111)	-0.53	2.93	-0.53	2.94	-0.44	3.10	-0.44	3.08
Cu(111)-Ni(25%)	-0.56	2.87	-0.56	2.89	-0.46	3.09	-0.46	3.09
Cu(111)-Ni(50%)-o/p	-0.58	2.84	-0.58	2.84	-0.48	3.01	-0.48	3.03
Cu(111)-Ni(75%)	-0.59	2.79	-0.59	2.79	-0.49	2.98	-0.49	2.98
Cu(111)-Ni(100%)	-0.61[b]/-0.80[a]	2.71[b]/2.09[a]	-0.62[b]/-0.80[a]	2.70[b]/2.09[a]	-0.50	2.95	-0.50	2.95
Ni(111)	-0.65	2.14	-0.64	2.14	-0.42	3.01	-0.42	3.01
Ni(111)-Cu(25%)	-0.59	$2.15^{Ni}/2.41^{Cu}$	-0.58	$2.15^{Ni}/2.42^{Cu}$	-0.43	$2.92^{Cu}/3.03^{Ni}$	-0.42	$2.95^{Cu}/3.05^{Ni}$
Ni(111)-Cu(50%)-o/p	-0.50	$2.88^{Cu}/2.90^{Ni}$	-0.50	$2.89^{Cu}/2.91^{Ni}$	-0.42	$2.98^{Cu}/3.07^{Ni}$	-0.42	$2.98^{Cu}/3.07^{Ni}$
Ni(111)-Cu(75%)	-0.49	2.92	-0.49	$2.92^{Cu}/2.93^{Ni}$	-0.42	$3.01^{Cu}/3.08^{Ni}$	-0.41	$3.02^{Cu}/3.10^{Ni}$
Ni(111)-Cu(100%)	-0.47	2.96	-0.47	2.96	-0.40	3.06	-0.40	3.07

[a, b] There are two stable adsorption configurations for $N_{top}B_{fcc}$ and $N_{top}B_{hcp}$ when monolayer h-BN on the Cu(111)-Ni surface alloy with a concentration of 100% Ni.

Table S2. The interaction energy ($\Delta E_{BN/M}$, $\Delta E_{G/BN}$, eV/supercell) and interlayer vertical distances (d_{BN-M}, d_{G-BN}, Å) of h-BN/graphene on the Cu-Ni alloy substrates with four different configurations. Adsorptiom configurations correspond to the illustration in Figure 1b.

Substrate	Stacking	1L-Graphene							
		$N_{top}B_{hollow}$(G/BN)				$B_{top}N_{hollow}$(G/BN)			
		$\Delta E_{BN/M}$	d_{BN-M}	$\Delta E_{G/BN}$	d_{G-BN}	$\Delta E_{BN/M}$	d_{BN-M}	$\Delta E_{G/BN}$	d_{G-BN}
h-BN/Cu(111)	$N_{top}B_{fcc}$	-0.54	2.90	-0.22	3.27	-0.55	2.90	-0.28	3.11
	$B_{top}N_{fcc}$	-0.39	3.06	-0.21	3.33	-0.46	3.04	-0.29	3.12
h-BN/Cu(111)-Ni(25%)	$N_{top}B_{fcc}$	-0.51	2.85	-0.21	3.33	-0.58	2.83	-0.28	3.11
	$B_{top}N_{fcc}$	-0.40	3.07	-0.20	3.32	-0.48	3.03	-0.28	3.13
h-BN/Cu(111)-Ni(50%)-o/p	$N_{top}B_{fcc}$	-0.52	2.80	-0.21	3.32	-0.59	2.79	-0.28	3.12
	$B_{top}N_{fcc}$	-0.42	3.00	-0.20	3.32	-0.49	2.98	-0.27	3.12
h-BN/Cu(111)-Ni(75%)	$N_{top}B_{fcc}$	-0.54	2.76	-0.21	3.31	-0.62	2.70	-0.29	3.10
	$B_{top}N_{fcc}$	-0.43	2.97	-0.20	3.31	-0.49	2.96	-0.27	3.14
h-BN/Cu(111)-Ni(100%)	$N_{top}B_{fcc}$	-0.82	2.07	-0.47	3.12	-0.86	2.07	-0.47	3.13
	$B_{top}N_{fcc}$	-0.45	2.92	-0.21	3.30	-0.51	2.90	-0.28	3.13
h-BN/Ni(111)	$N_{top}B_{fcc}$	-0.80	2.12	-0.36	3.11	-0.79	2.13	-0.63	3.12
	$B_{top}N_{fcc}$	-0.46	2.99	-0.31	3.23	-0.52	3.00	-0.36	3.10
h-BN/Ni(111)-Cu(25%)	$N_{top}B_{fcc}$	-0.67	$2.14^{Ni}/2.35^{Cu}$	-0.64	3.05	-0.73	$2.14^{Ni}/2.34^{Cu}$	-0.70	3.08
	$B_{top}N_{fcc}$	-0.45	$2.93^{Cu}/3.04^{Ni}$	-0.61	3.26	-0.52	$2.92^{Cu}/3.02^{Ni}$	-0.67	3.11
h-BN/Ni(111)-Cu(50%)-o/p	$Nt_{op}B_{fcc}$	-0.56	$2.25^{Ni}/2.55^{Cu}$	-0.58	3.13	-0.64	$2.24^{Ni}/2.51^{Cu}$	-0.65	3.05
	$B_{top}N_{fcc}$	-0.45	$2.96^{Cu}/3.05^{Ni}$	-0.58	3.26	-0.52	$2.95^{Cu}/3.05^{Ni}$	-0.65	3.10
h-BN/Ni(111)-Cu(75%)	$N_{top}B_{fcc}$	-0.53	$2.90^{Cu}/2.92^{Ni}$	-0.56	3.24	-0.59	$2.89^{Cu}/2.91^{Ni}$	-0.71	3.11
	$B_{top}N_{fcc}$	-0.44	$3.01^{Cu}/3.09^{Ni}$	-0.56	3.27	-0.51	$3.00^{Cu}/3.08^{Ni}$	-0.63	3.10
h-BN/Ni(111)-Cu(100%)	$N_{top}B_{fcc}$	-0.52	2.93	-0.31	3.24	-0.57	2.94	-0.36	3.10
	$B_{top}N_{fcc}$	-0.42	3.07	-0.29	3.27	-0.49	3.07	-0.35	3.11

Table S3. The interaction energy (ΔE_{BN-M}, ΔE_{G-BN}, ΔE_{G1-G2}, eV/supercell) and interlayer vertical distance (d_{BN-M}, d_{G-BN}, d_{G1-G2},Å) of the 2L-Gr/h-BN/Cu(111)-Ni on the Cu-Ni alloy substrates with the $B_{top}N_{hollow}$ABA configurations. Adsorptiom configurations correspond to the illustration in Figure 1b.

Substrate stacking	2L-Graphene					
	$B_{top}N_{hollow}$ABA					
$N_{top}B_{fcc}$	ΔE_{BN-M}	d_{BN-M}	ΔE_{G-BN}	d_{G-BN}	ΔE_{G1-G2}	d_{G1-G2}
h-BN/Cu(111)	-0.52	2.91	-0.24	3.12	-0.18	3.26
h-BN/Cu(111)-Ni(25%)	-0.54	2.82	-0.29	3.09	-0.19	3.26
h-BN/Cu(111)-Ni(50%)-o/p	-0.56	2.78/2.79	-0.30	3.12	0.19	3.26
h-BN/Cu(111)-Ni(75%)	-0.58	2.70/2.71	-0.30	3.10	0.19	3.27
h-BN/Cu(111)-Ni(100%)	-0.83	2.08	-0.34	3.12	0.19	3.27
h-BN/Ni(111)	-0.88	2.13	-0.48	3.12	-0.28	3.24
h-BN/Ni(111)-Cu(25%)	-0.79	$2.14^{Ni}/2.34^{Cu}$	-0.51	3.08	-0.28	3.22
h-BN/Ni(111)-Cu(50%)-o/p	-0.69	$2.24^{Ni}/2.51^{Cu}$	-0.50	3.05	-0.28	3.23
h-BN/Ni(111)-Cu(75%)	-0.64	$2.89^{Cu}/2.91^{Ni}$	-0.46	3.11	-0.28	3.24
h-BN/Ni(111)-Cu(100%)	-0.63	2.93	-0.46	3.10	-0.28	3.24

Table S4. The total energy (E_{total}, eV), interaction energy (ΔE, eV/supercell) and interlayer vertical distance (d_{BNG}, Å) of free Gr/h-BN heterostructures with different stacking.

Systems	Stacking		$B_{top}N_{hollow}$	$N_{top}B_{hollow}$	$B_{top}N_{top}$
1L-G/h-BN		d_{BNG}	3.15	3.31	3.38
		ΔE_{BNG}	-0.26	-0.22	-0.20
		E_{total}	-36.80	-36.74	-36.72
2L-G/h-BN	AA	d_{BNG}	3.13	3.29	3.37
		d_{G1G2}	3.50	3.51	3.53
		ΔE_{BNG}	-0.64	-0.20	-0.18
		ΔE_{G1G2}	-0.36	-0.12	-0.10
		E_{total}	-55.65	-55.59	-55.57
	AB	d_{BNG}	3.12	3.31	3.38
		d_{G1G2}	3.27	3.27	3.24
		ΔE_{BNG}	-0.32	-0.26	-0.24
		ΔE_{G1G2}	-0.24	-0.16	-0.16
		E_{total}	-55.71	-55.64	-55.63
3L-G/h-BN	ABA	d_{BNG}	3.12	3.31	3.37
		d_{G1G2}	3.25	3.27	3.26
		d_{G2G3}	3.30	3.27	3.26
		ΔE_{BNG}	-0.32	-0.26	-0.24
		ΔE_{G1G2}	-0.28	-0.22	-0.20
		ΔE_{G2G3}	-0.22	-0.16	-0.16
		E_{total}	-74.61	-74.55	-74.54
	ABC	d_{BNG}	3.12	3.31	3.42
		d_{G1G2}	3.25	3.27	3.26
		d_{G2G3}	3.27	3.28	3.28
		ΔE_{BNG}	-0.32	-0.26	-0.24
		ΔE_{G1G2}	-0.28	-0.22	-0.20
		ΔE_{G2G3}	-0.24	-0.16	-0.14
		E_{total}	-74.62	-74.55	-74.53

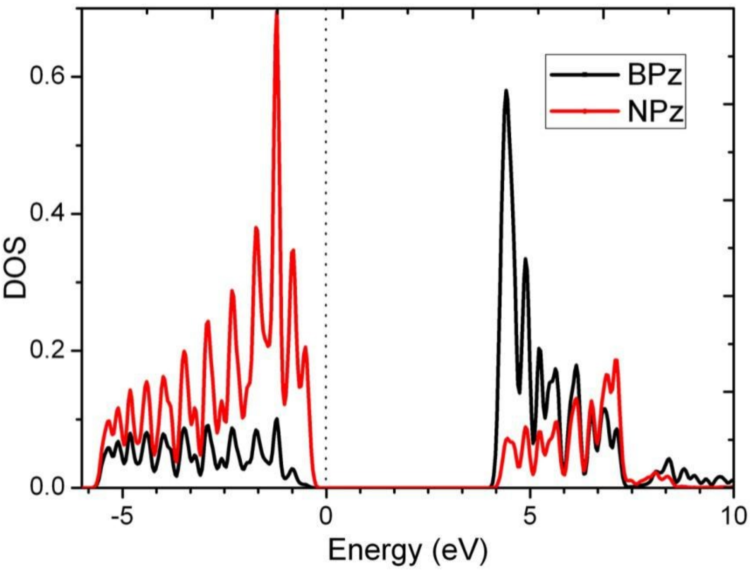

Figure S1. Partial density of states of B and N atoms for free monolayer h-BN.

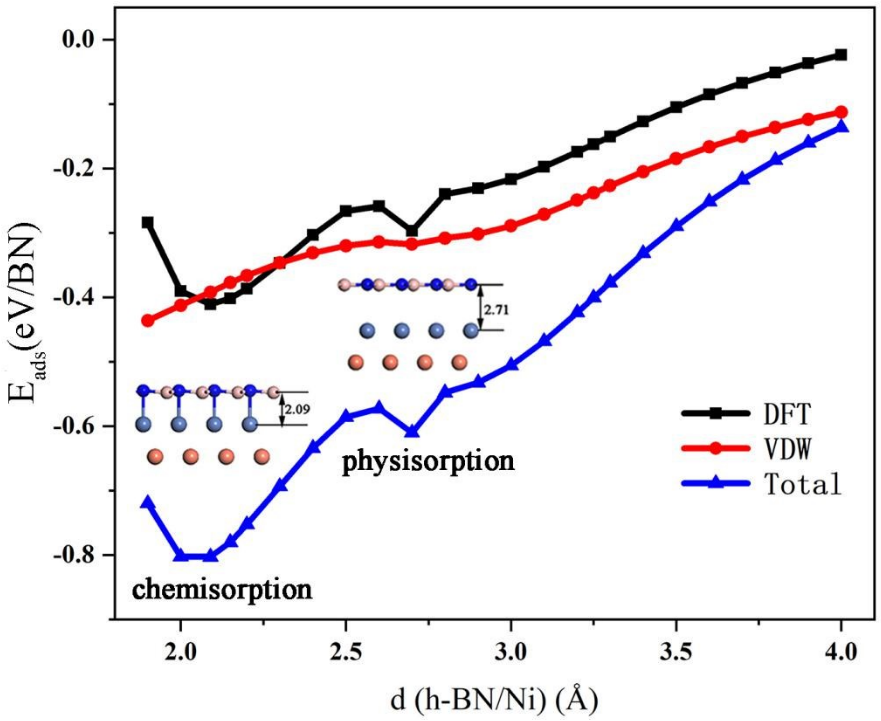

Figure S2. Binding energy of h-BN adsorbed on Cu(111)-Ni(100%) in different adsorption geometries versus distance between h-BN and Cu(111)-Ni(100%) surface. Total binding energies are displayed as a blue line, DFT and vdW contributions to the binding energy as black and red lines, respectively.

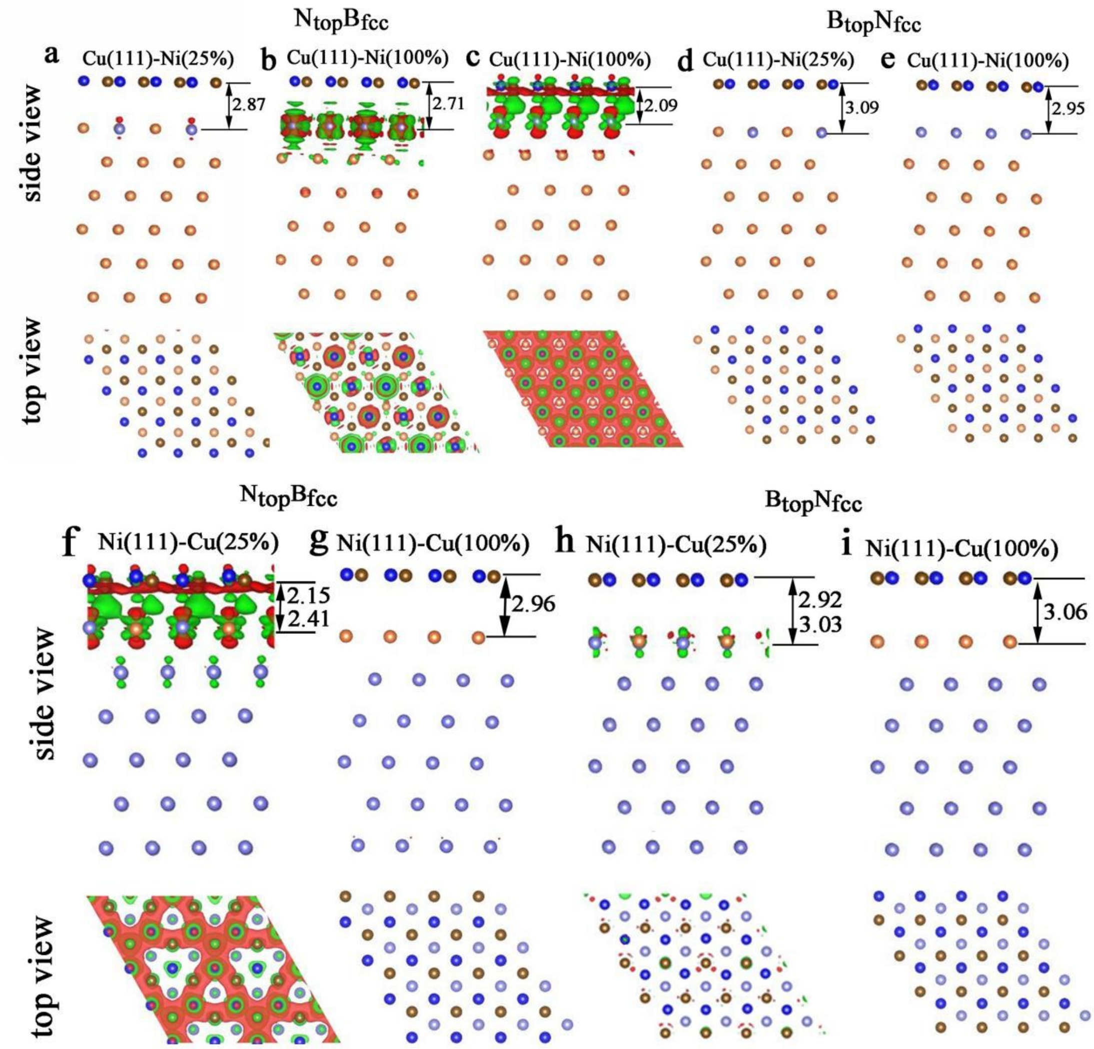

Figure S3. Charge density difference plots of the h-BN on Cu(111)-Ni(25%, 100%), Ni(111)-Cu(25%, 100%) with different geometries. Here, the charge density difference refers to the variance between the total charge density of the h-BN/Cu(111)-Ni/Ni(111)-Cu and the sum of the charge density of the separated Cu and Ni layer and monolayer h-BN layer, which are kept the same geometric structures as those in the h-BN/ Cu(111)-Ni/Ni(111)-Cu. The red and green color regions mark the depletion and accumulation of electronic charges, respectively. The brown, blue, purple and orange represent B, N, Ni and Cu atoms, respectively.

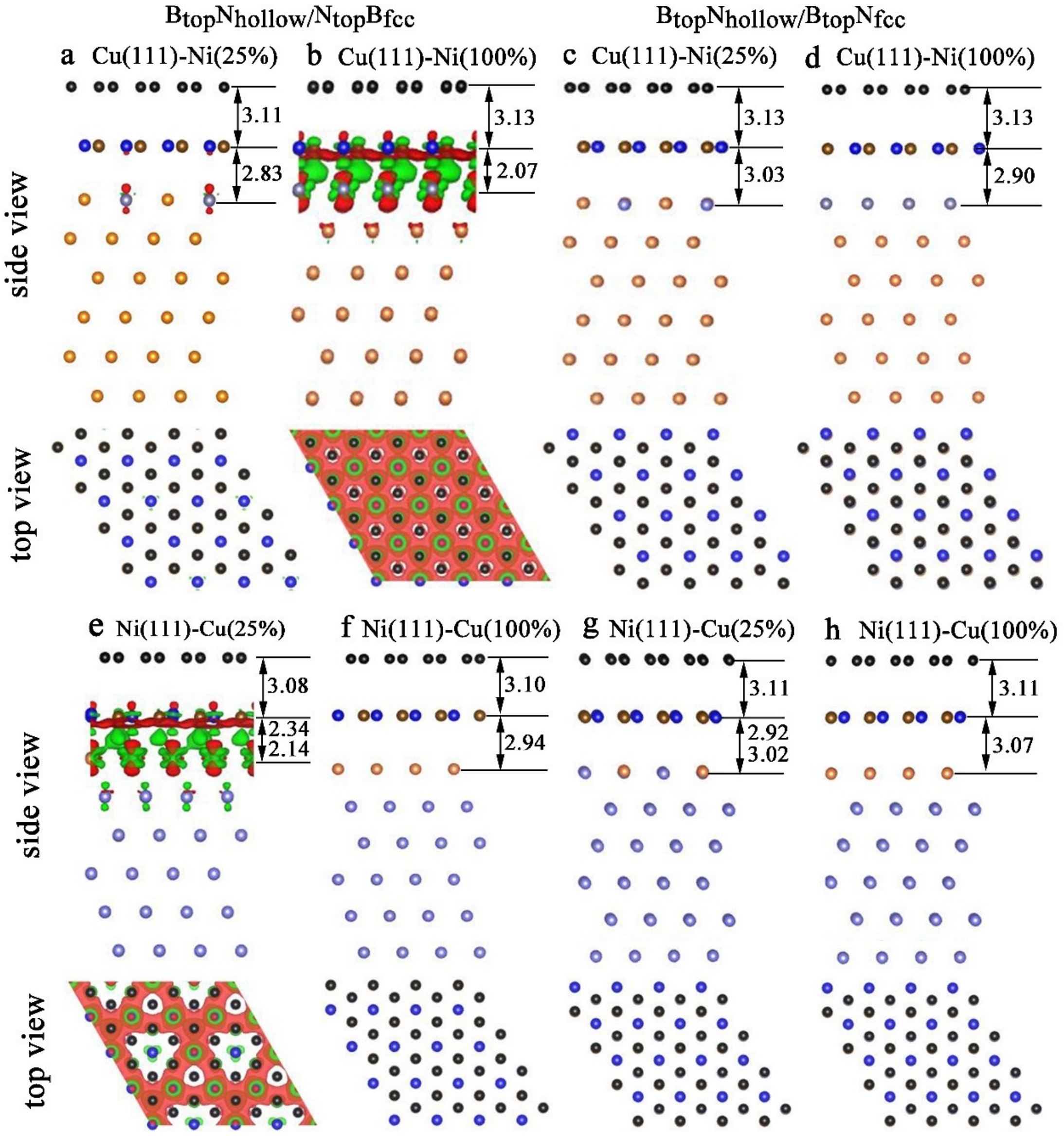

Figure S4. Charge density difference plots of monolayer graphene on h-BN/Cu-100%Ni alloy substrate with two different geometries. Here, the charge density difference refers to the variance between the total charge density of the graphene/h-BN/Cu-Ni alloy and the sum of the charge density of the separated graphene/h-BN and Cu-Ni alloy substrate. The red and green color regions mark the depletion and accumulation of electronic charges, respectively. The black, brown, blue, purple and orange represent C, B, N, Ni and Cu atoms, respectively.
